# Xylanases of *Cellulomonas flavigena*: expression, biochemical characterization, and biotechnological potential

**DOI:** 10.1186/s13568-016-0308-7

**Published:** 2017-01-03

**Authors:** Alexander V. Lisov, Oksana V. Belova, Zoya A. Lisova, Nataliy G. Vinokurova, Alexey S. Nagel, Zhanna I. Andreeva-Kovalevskaya, Zhanna I. Budarina, Maxim O. Nagornykh, Marina V. Zakharova, Andrey M. Shadrin, Alexander S. Solonin, Alexey A. Leontievsky

**Affiliations:** 1 G.K. Skryabin Institute of Biochemistry and Physiology of Microorganisms, Russian Academy of Sciences (IBPM RAS), 5 Prospekt Nauki, Pushchino, Moscow Region 142290 Russia; 2 Pushchino State Institute of Life Sciences, 3 Prospekt Nauki, Pushchino, Moscow Region 142290 Russia

**Keywords:** Xylanases, *Cellulomonas flavigena*, Xylooligosaccharides, Saccharification of cereals

## Abstract

Four xylanases of *Cellulomonas flavigena* were cloned, expressed in *Escherichia coli* and purified. Three enzymes (CFXyl1, CFXyl2, and CFXyl4) were from the GH10 family, while CFXyl3 was from the GH11 family. The enzymes possessed moderate temperature stability and a neutral pH optimum. The enzymes were more stable at alkaline pH values. CFXyl1 and CFXyl2 hydrolyzed xylan to form xylobiose, xylotriose, xylohexaose, xylopentaose, and xylose, which is typical for GH10. CFXyl3 (GH11) and CFXyl4 (GH10) formed the same xylooligosaccharides, but xylose was formed in small amounts. The xylanases made efficient saccharification of rye, wheat and oat, common components of animal feed, which indicates their high biotechnological potential.

## Introduction

Hemicelluloses are polysaccharides which, along with cellulose and lignin, are the main polymer components of a plant cell wall. According to sugars, polysaccharide monomers, hemicelluloses are divided into xyloglucans, xylans, mannans, glucomannans, arabinoxylans, and β-glucans (Scheller and Ulvskov 2010). The most common hemicellulose is xylan. This polysaccharide consists of the β-(1-4) linked xylose backbone with a small amount of β-(1-3) branches, often with *O*-acetyl groups (Ebringerova and Heinze 2000). Xylan occurs among terrestrial plants and green algae. Xylan as a carbon source can be utilized by many bacteria and fungi. To destroy xylan, microorganisms produce several enzymes. The main role in the destruction of xylan is played by xylanase (endo-1,4-β-xylanase, E.C. 3.2.1.8), which catalyzes the random hydrolysis of xylan to xylooligosaccharides. Another enzyme, β-xylosidase (xylan-1,4-β-xylosidase, E.C. 3.2.1.37), completes the process of decomposition by releasing xylose residues from the nonreducing ends of xylooligosaccharides. The side chains and acyl groups of the xylan are cleaved by two enzymes: glucuronidase (α-glucosiduronase, E.C. 3.2.1.139) and acetylxylan esterase (E.C. 3.1.1.72) (Subramaniyan and Prema 2002). According to the classification of hydrolases, which is based on the similarity of their primary structures and is described in Carbohydrate-Active Enzyme (CAZy) database (http://www.cazy.org), xylanases belong to different glycoside hydrolase (GH) families. The xylanases of GH10 and GH11 families have been studied most comprehensively (Motta et al. 2013). Xylanases of GH10 have a molecular weight of around 40–60 kDa and exhibit catalytic versatility: they act not only on xylan but also on arabinoxylan. Xylanases of GH11 possess a lower molecular weight and act only on xylan. Producers of GH10 and GH11 xylanases are widespread among fungi and bacteria (Subramaniyan and Prema 2002). Xylanases are actively used in practice to improve the properties of animal feed, in the baking industry, and for bleaching paper pulp (Harris and Ramalingam 2010; Beg et al. 2001). Due to practical importance of the enzyme, a search for new efficient producers of xylanases is carried.

*Cellulomonas flavigena* is a Gram-positive bacterium, which possesses high cellulolytic and xylolytic activities. For the decomposition of polysaccharides, the bacterium produces a large number of hydrolases (Sánchez-Herrera et al. 2007). Some hydrolases from *C. flavigena*, including xylanases, were obtained in a purified state, and their biochemical properties and biotechnological potential were studied (Pérez-Avalos et al. 2008; Amaya-Delgado et al. 2010). Therefore, this bacterium might be a promising source of xylanases. This was confirmed by the data of the *C. flavigena* genome sequence, where 14 genes potentially encoding xylanases were found (Abt et al. 2010). The presence of the whole genome sequence also facilitates genetic manipulations with this bacterium. The article describes the production of recombinant xylanases of *C. flavigena* and the study of their properties and biotechnological potential.

## Materials and methods

### Cultivation of microorganism, cloning, recombinant expression and purification of xylanases

The strain *C. flavigena* Ac-1137 was obtained from the All-Russian Collection of Microorganisms (VKM, http://vkm.ru). The strain was grown on a medium containing (g/l of tap water): peptone 5.0; yeast extract 3.0; KH_2_PO_4_ 0.2; glucose 5.0; pH 7.2. The genomic DNA was purified from biomass using the Genomic DNA Purification Kit (Fermentas). Primers for PCR (Table 1) were constructed based on the GH10 and GH11 endo-1,4-β–xylanase sequences from the genome of *C. flavigena* DSM 20109 (NCBI Reference Sequence of Genome: NC_014151.1). The DNA fragments encoding xylanases were amplified by the PCR technique with primers. PCR, cloning, expression and purification of the proteins were performed as previously described (Lisov et al. 2014). Q5 DNA-polymerase (NEB) was used for the xylanases genes amplification.

**Table 1:** Primers used for PCR amplification of the xylanases genes

NCBI reference sequence	Forward primer	Reverse primer
WP_013115499.1	GGTACCGGATCCCAGAACGTCAGCAGC	CTGCAGAAGCTTTCAGGAGCAGATGCC
WP_013115627.1	GTACCGGATCCGCTCCCGCTCACG	CTGCAGAAGCTTTCACCCGACCTTCACG
WP_043598780.1	CGCGGATCCGCGGTCGCCGAGAC	CCCAAGCTTTCACGACCTCGGCCTG
WP_013118747.1	CGCGGATCCACGTCCCCCACGCC	CCCAAGCTTTTACTCGCCGGCCAGC
WP_013118731.1	CGCGGATCCGCGGGCAGCACGC	CCCAAGCTTTCAGGAGGCCGTGCAG
WP_013117551.1	TACGGATCCGCGCCGGGCTGGTC	CTGCAAGCTTTCAGCGCGGCCGCG
WP_013117277.1	TACGGATCCATGACCGCCCAGCCGATC	CTGCAAGCTTTCATCGCGCGGCCACG
WP_013118238.1	TACGGATCCGCGGAGAGCACGCTCG	CTGCAAGCTTCTACCGCTGCAGCGTCA
WP_043598750.1	TACGGATCCGCGGACCCCGTGAGC	CTGCAAGCTTTCAGCGCCGCAGCAG
WP_052302667.1	TACGGTACCATGGTCGGGACGACCCTG	CTGCAAGCTTTCAGCGCCAGACGACCA

The presence of a signal peptide was determined using the SignalP 4.0 service (http://www.cbs.dtu.dk/services/SignalP/). The presence of conserved domains was determined using the InterPro Scan (http://www.ebi.ac.uk/Tools/pfa/iprscan/) and BLAST (http://blast.ncbi.nlm.nih.gov/Blast.cgi) software.

### Enzyme characterization

The xylanase activity was assayed by measuring the amount of reducing sugars produced from beechwood xylan (Sigma) by the ferricyanide method (Friedemann et al. 1962) using xylose as the standard. The reaction mixture consisted of 1% xylan in 75 mM of universal buffer, pH 7.0. Typically, the reaction mixture consisted of 20 μl of the enzyme and 300 μl of the xylan solution. The mixture was incubated at 40 °C for 10 min. One unit of xylanase activity was defined as the amount of enzyme that forms reducing groups corresponding to 1 μmol of xylose in 1 min under the above conditions. Reactions with other substrates were done in the same conditions, but with replacement of xylan by 1% CM-cellolose or microcrystalline cellulose or barley β-glucan. The protein concentration was determined using the molar absorption coefficient at 280 nm calculated from the protein sequence.

Characterization of properties of xylanases was performed as previously described (Lisov et al. 2014).

The molecular weight of purified proteins was determined by SDS-PAGE using 12% gel according to Laemmly (1970). The standard proteins were as follows: beta-galactosidase (116 kDa), bovine serum albumin (66 kDa), ovalbumin (45 kDa), lactate dehydrogenase (35 kDa), REase Bsp98I (25 kDa), beta-lactoglobulin (18.4 kDa), and lysozyme (14.4 kDa).

### Enzymatic hydrolysis of beechwood xylan and saccharification experiments

The hydrolysis of beechwood xylan was performed in 75 mM Britton–Robinson buffer, pH 7.0, at 40 °C with the xylan concentration of 2% and the enzyme activity of 2.5 U/ml. At definite intervals, samples were taken from the reaction mixture, and the reaction in the selected samples was stopped by boiling for 5 min. Samples an enzyme inactivated by 10-min boiling were incubated for 22 h and used as controls. The hydrolysis products were analyzed by TLC using an HPTLC Silica gel 60 plate (Merck) and a butanol–acetic acid–diethyl ether–water (9:6:3:1) eluting system. Then the plate was dried at 105 °C and sprayed with a mixture containing 1% diphenyl amine, 1% aniline, and 3% phosphoric acid in acetone. After spraying, the plate was kept at 120° for 10 min. Xylose was applied onto the plate as the standard. Other hydrolysis products (xylobiose—X2, xylotriose—X3, xylotetraose—X4, xylopentaose—X5, xylohexaose—X6) were eluted from the plate with methanol and identified by an LCQ Advantage MAX tandem mass spectrometer (Thermo Finnigan).

Wheat, oat, and rye were used for experiments with saccharification of xylan-containing materials. Experiments were done as described previously (Lisov et al. 2014) except that the 75 mM universal buffer was used with pH optimum of the xylanases (i.e. pH 7.0 for CFXyl1 and CFXyl3, and pH 7.5 for CFXyl2 and CFXyl4). The amount of reducing sugars formed was determined using the ferricyanide method.

## Results

**Fig. 1:**
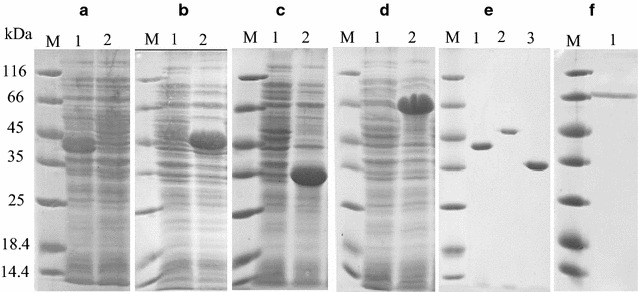
The electrophoretic study of the production of xylanases (**a**–**d**) and results of their purification (**e**–**f**). **a** CFXyl1: *1*—with IPTG, *2*—without IPTG; **b** CFXyl2: *1*—without IPTG, *2* —with IPTG; **c** CFXyl3: *1*—without IPTG, *2*—with IPTG; **d** CFXyl4: *1*—without IPTG, *2*—with IPTG; **e** Purified enzymes: *1*—CFXyl1; *2*—CFXyl2; *3*—CFXyl3. **f** Purified CFXyl4. *M* molecular weight markers

In the genome of *C. flavigena,* nine genes of GH10 xylanase (NCBI Reference Sequences WP_013115627.1; WP_043598750.1; WP_052302667.1; WP_013118747.1; WP_013118731.1; WP_013117551.1; WP_043598780.1; WP_013117277.1; WP_013118238.1) and one gene of GH11 xylanase (NCBI Reference Sequence WP_013115499.1) were found. The genes encoding the xylanases were obtained by the PCR. PCR products of were cloned into pQE-30 vector. We failed to obtain the clones of three genes: of proteins WP_052302667.1, WP_043598750.1, and WP_013118238.1. The sequences of the other cloned genes were completely identical to those deposited in GenBank. After the transformation of *E. coli* with pQE-30 plasmid, the xylanase production was investigated. The genes of four proteins were expressed: WP_013118747.1, WP_013115627.1, WP_013115499.1, and WP_043598780.1, which were called CFXyl1, CFXyl2, CFXyl3, and CFXyl4, respectively (Fig. 1a–c). The sequences of CFXyl1, CFXyl2, CFXyl4 contained the GH10 domain, while CFXyl3 contained the GH11 domain. The sequences of the proteins had the TAT signal peptide, indicating that they all are secreted proteins. The part of the CFXyl3 sequence from 257 to 343 amino acids is a carbohydrate-binding domain of the CBM2 family, and the CFXyl2 sequence from 354 to 492 amino acids contains the Ricin B-like lectin domain, which has carbohydrate-recognizing properties (Boraston et al. 2004). So, this indicates that CFXyl2 and CFXyl3 possess the carbohydrate-binding activity.

The use of pQE-30 enables one to produce N-terminal 6His-tagged recombinant proteins. All recombinant proteins were synthesized as mature proteins without the TAT signal peptide. To avoid the formation of inclusion bodies at the induction stage, the induction was carried out at low temperature and aeration, as well as low concentrations of IPTG. After a two-stage purification, the enzymes were obtained in an electrophoretically homogeneous state (Fig. 1e, f) with yields of 66, 40.1, 50.5, and 45% for CFXyl1, CFXyl2, CFXyl3, and CFXyl4, accordingly. The molecular weight was 44 kDa for CFXyl1, 53 kDa for CFXyl2, 38 kDa for CFXyl3, and 73 kDa for CFXyl4, which is in good agreement with the molecular weights, calculated from the proteins sequences. The enzymes did not catalyze the hydrolysis of CM-cellulose, microcrystalline cellulose, and barley β-glucan. All enzymes catalyzed the hydrolysis of xylan.

**Fig. 2:**
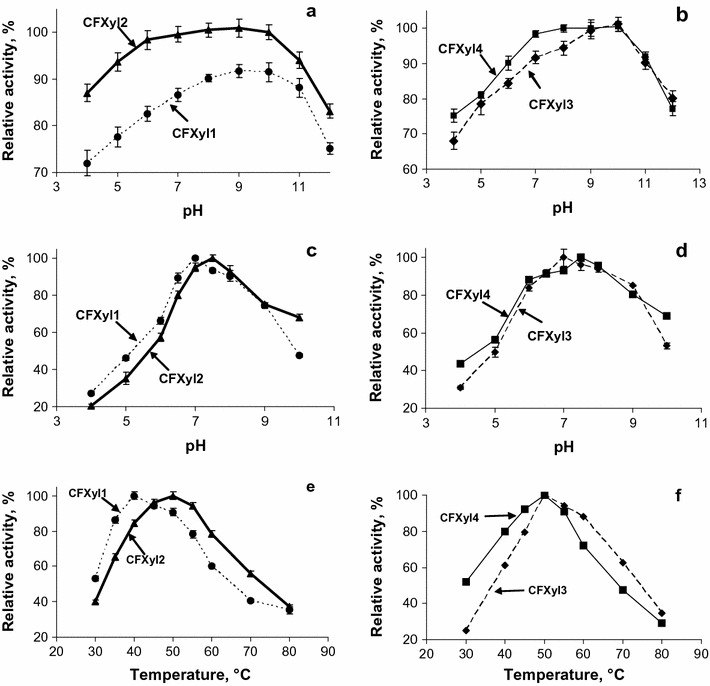
Properties of CFXyl1–CFXyl4 xylanases. **a**, **b** pH stability of the xylanases; **c**, **d** pH optima of the xylanases; **e**, **f** temperature optimum. *Dotted line* CFXyl1; *bold line* CFXyl2; *dashed line* CFXyl3; *solid line* CFXyl4

**Fig. 3:**
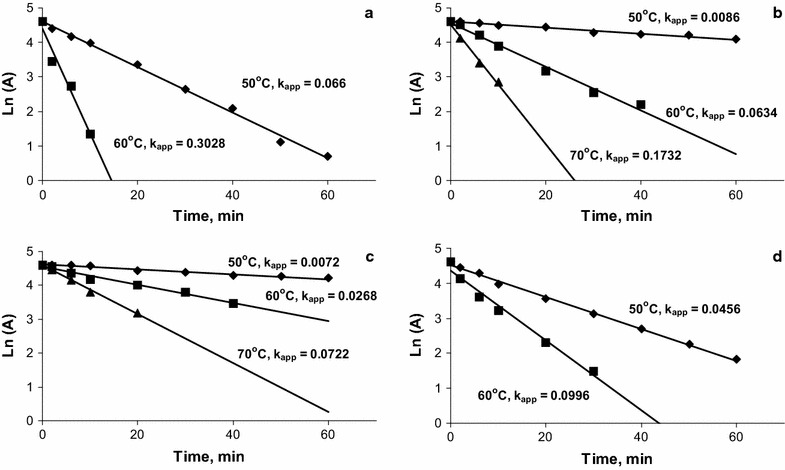
Thermal stability of CFXyl1–CFXyl4 xylanases. Ln (**a**) the natural logarithm of the residual activity. **a** CFXyl1; **b** CFXyl2; **c** CFXyl3; **d** CFXyl4

The pH optimum of the activity of xylanases was neutral (7.0–7.5) (Fig. 2c, d). The xylanases were active in the pH range 4–10 and retained more than 50% of the activity at pH 5.0–9.0. The enzymes were more stable at slightly alkaline pH; as the pH was lowered, the enzyme stability decreased and at pH more than 11 stability was strongly reduced (Fig. 2a, b). The temperature optimum for the hydrolysis of xylan by CFXyl1 was 40 °C, while the optimum for CFXyl2, CFXyl3 and CFXyl4 was 50 °C (Fig. 2e, f). CFXyl1 and CFXyl4 showed a lesser thermostability than CFXyl3 and CFXyl2 (Fig. 3). The half-lives for CFXyl1 and CFXyl4 at 60 °C were 2.3 and 7 min, and at 70 °C the enzymes were inactivated rapidly. CFXyl3 and CFXyl1 were active at 70 °C. The half-life time of CFXyl3 was 9.6 min at this temperature; thus, its stability was the highest among the four enzymes.

**Fig. 4:**
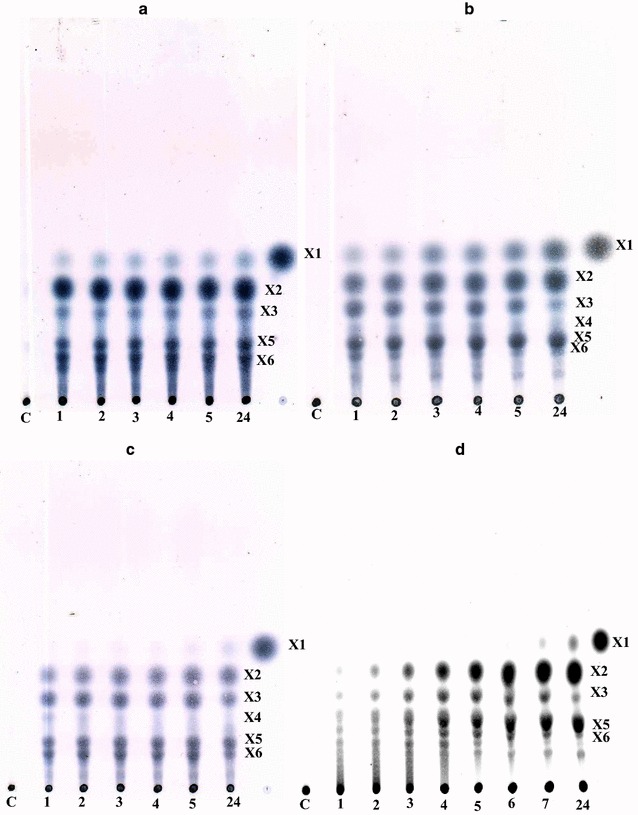
Products of xylan hydrolysis by: **a** CFXyl1; **b** CFXyl2; **c** CFXyl3; **d** CFXyl4. Time of reaction is indicated by numbers in hours. *C* control. Xylooligosaccharides: *X1* xylose, *X2* xylobiose, *X3* xylotriose, *X4* xylotetraose, *X5* xylopentaose, *X6* xylohexaose

Under the action of CFXyl1 and CFXyl2, the main products of xylan hydrolysis were xylobiose and xylopentaose (Fig. 4a, b). CFXyl1 produced xylose as the main product, whereas CFXyl2 formed xylose to a lesser extent. In both cases, xylose appeared at the start of the reaction. Xylotriose and xylohexose were the minor products of hydrolysis for both enzymes. In the case of CFXyl1, at the initial stage of the reaction, the formation of xylotetraose was observed, which disappeared during further hydrolysis. Xylotetraose also was the reaction product of CFXyl3; it also disappeared during the reaction (Fig. 4c). The main products of xylan hydrolysis by CFXyl3 were xylobiose, xylotriose, xylopentaose, and xylohexaose. A small amount of xylose formed after several hours of reaction. The main products of xylan hydrolysis by CFXyl4 were xylobiose and xylopentaose; in addition, xylotriose and xylohexaose formed, though in much smaller amounts (Fig. 4d). Xylose, like CFXyl3, formed in small amounts after some hours of reaction.

**Fig. 5:**
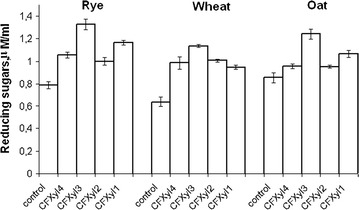
Formation of reducing sugars from rye, wheat and oat under the action of CFXyl1, CFXyl2, CFXyl3 and CFXyl4

The xylanases hydrolyzed polysaccharides of rye, wheat, and oats, which are the common animal feed ingredients. All xylanases significantly enhanced reducing sugar content compared to basal levels (control). The enzymes saccharified cereals with different efficiency. CFXyl3 showed the highest efficacy, while CFXyl4 was the least effective (Fig. 5). CFXyl1 and CFXyl2 hydrolyzed cereals with a lower efficiency than CFXyl3 but more efficiently than CFXyl4. When CFXyl1 hydrolyzed oat and rye, the amount of reducing sugars was greater than that hydrolyzed by CFXyl2. CFXyl2 hydrolyzed wheat better than CFXyl1 (Fig. 5).

## Discussion

Bacteria of the genus *Cellulomonas* are of great interest, since they produce large amounts of enzymes important for biotechnology. Therefore, several genomes of *Cellulomonas* were sequenced (Weiping Zhuang et al. 2015). These bacteria are active producers of xylanases. A comparison of literature data on the properties of *Cellulomonas* xylanases with the data of this study showed that the xylanases of these bacteria are moderately thermally stable (Hekmat et al. 2005; Chaudhary and Deobagkar 1997; Amaya-Delgado et al. 2010). The enzymes possess good stability at temperatures below 60 °C and are rapidly inactivated at 65–70 °C. As shown in the present article, some *C. flavigena* strains have xylanases with different thermostability. The optimum pH of their activity was close to neutral values, from 5.7 in *C. fimi* (Chen et al. 2012) to 6.5 in *C. flavigena* (Amaya-Delgado et al. 2010; Santiago-Hernández et al. 2007). There are data on xylanase from *C. fimi* with a weakly acidic pH optimum, 5.0 (Sunil Khanna and Gauri 1993). Xylanases were most stable at pH 8–10. But the pH optimum was neutral. Typically, the pH optimum correlates with pH stability, although in many proteins such correlation was not observed, because pH optimum is most important for the adaptation of enzymes to the biophysical characteristics of the corresponding environment (Talley and Alexov 2010). The temperature optimum of *Cellulomonas* xylanases is within 50–60 °C, which is probably due to the moderate thermostability of the enzymes. Xylanase Cflxyn11A of GH11 from *C. flavigena* was previously described (Amaya-Delgado et al. 2010). In the present study, the only GH11 xylanase (CFXyl3) was found in the genome of *C. flavigena,* and its identity with Cflxyn11A was 66%. It is possible that there exists a difference between *C. flavigena* strains in the sequence of GH11 xylanases, or in some strains there may be more than one GH11 gene.

Xylanases hydrolyze xylan to xylooligosaccharides. It is believed that the xylanases of GH11 and GH10 families hydrolyze xylan with different specificity (Biely et al. 1997). GH10 xylanases hydrolyze xylan to low-molecular-weight products, and xylose is one of the main products of hydrolysis. GH11 xylanases cleave internal β-1,4-xylosidic bonds of polymeric xylan and are less active toward xylooligosaccharides. The basis for the difference in specificity is the structural features of the GH11 and GH10 families (Pollet et al. 2010). Indeed, there is evidence for the correctness of this approach; differences in the products of xylan hydrolysis between GH11 and GH10 are known (Ustinov et al. 2008; Zhenhua Qiua et al. 2010). The xylanases of *C. flavigena* correspond to this regularity: the products of xylan hydrolysis by CFXyl1 and CFXyl2 were of low molecular weight, including xylose, while the CFXyl3 formed very small amounts of xylose In contrast to majority of GH10 family members, CFXyl4 xylanase, like GH11 xylanases, produced very small amounts of xylose. Probably, not all GH10 xylanases form xylose as the main hydrolysis product of xylan, and some members of the GH10 family are structurally similar to GH11 xylanases. GH10 xylanases that did not form xylose were described previously (Waeonukul et al. 2009; Zhang et al. 2010).

Xylanases are actively used to improve the nutritional quality of animal feed. They increase the productivity of monogastric animals (He et al. 2010; Pirgozliev et al. 2010). CFXyl1–CFXyl4 hydrolyzed grains with different efficiency, which may be due to the effect of xylanase inhibitors on xylanases. The inhibitors are widely distributed among the cereals (Gebruers et al. 2010). It is also possible that the difference is associated with the effect of lignin contained in cereals, which is known to have an inhibitory effect on xylanases (Berlin et al. 2006). Nevertheless, CFXyl1–CFXyl4 efficiently hydrolyzed substrates, indicating their high potential for improving the properties of animal feed. CFXyl3 revealed the greatest activity. It is more suitable for the use in biotechnology than the others, taking the higher thermal stability and neutral pH optimum of this enzyme into account.

## Abbreviations

VKM: All-Russian Collection of Microorganisms
PCR: polymerase chain reaction
LB: Luria and Bertani medium
IPTG: isopropyl-β-thiogalactopyranoside
SDS-PAGE: sodium dodecyl sulfate polyacrylamide gel electrophoresis
DNA: deoxyribonucleic acid
